# DnaK3 Is Involved in Biogenesis and/or Maintenance of Thylakoid Membrane Protein Complexes in the Cyanobacterium *Synechocystis* sp. PCC 6803

**DOI:** 10.3390/life10050055

**Published:** 2020-04-30

**Authors:** Adrien Thurotte, Tobias Seidel, Ruven Jilly, Uwe Kahmann, Dirk Schneider

**Affiliations:** 1 Department of Chemistry, Biochemistry, Johannes Gutenberg University Mainz, 55128 Mainz, Germany; adrienthurotte@netcourrier.com (A.T.); TobiasSeidel@gmx.net (T.S.); ruvenjilly@gmail.com (R.J.); 2 Institute of Molecular Biosciences, Goethe University Frankfurt, Max-von-Laue Straße 9, 60438 Frankfurt, Germany; 3 Department of Molecular Cell Biology, Bielefeld University, 33615 Bielefeld, Germany; ZUD@gmx.de

**Keywords:** chaperone, Hsp70, photosynthesis, thylakoid membrane biogenesis, photosystem maintenance, *Synechocystis* sp. PCC6803

## Abstract

DnaK3, a highly conserved cyanobacterial chaperone of the Hsp70 family, binds to cyanobacterial thylakoid membranes, and an involvement of DnaK3 in the biogenesis of thylakoid membranes has been suggested. As shown here, light triggers synthesis of DnaK3 in the cyanobacterium *Synechocystis* sp. PCC 6803, which links DnaK3 to the biogenesis of thylakoid membranes and to photosynthetic processes. In a DnaK3 depleted strain, the photosystem content is reduced and the photosystem II activity is impaired, whereas photosystem I is regular active. An impact of DnaK3 on the activity of other thylakoid membrane complexes involved in electron transfer is indicated. In conclusion, DnaK3 is a versatile chaperone required for biogenesis and/or maintenance of thylakoid membrane-localized protein complexes involved in electron transfer reactions. As mentioned above, Hsp70 proteins are involved in photoprotection and repair of PS II in chloroplasts.

## 1. Introduction

In plants and cyanobacteria, the biogenesis and dynamics of thylakoid membranes (TMs) is light-controlled [1,2]. In plants, proplastids develop into chloroplasts, involving the de novo formation of an internal TM network [3], and a developed TM network dynamically reorganizes in the light [4]. When the cyanobacterium *Synechocystis* sp. PCC 6803 (from here on: *Synechocystis*) is grown in the dark under light-activated heterotrophic growth (LAHG) conditions, where glucose is the only available energy source, *Synechocystis* cells exhibit reduced or even just rudimentary TMs [5,6]. However, after shifting dark-adapted cells into the light, the *Synechocystis* cells quickly rebuild a TM network and recover photosynthetic activity [5,7]. While dark-adapted *Synechocystis* cells do not harbor active photosystem II (PS II) complexes, complete photosynthetic activity is regained within 24 h after transferring dark-adapted cells into the light, and reappearance of photosynthetic electron transfer processes is coupled to the formation of internal TMs [7]. However, it is still enigmatic how the formation of internal TMs is controlled, both in chloroplasts and cyanobacteria, although some proteins that might be involved in this process have already been described previously [8]. These proteins include the inner membrane-associated protein of 30 kDa (IM30, also known as Vipp1: The vesicle-inducing protein in plastids 1), Hsp70 (Heat shock protein 70) chaperones, dynamin-like proteins, a prohibitin-like protein, as well as YidC, a membrane protein integrase [9,10,11,12,13,14,15,16]. Nevertheless, while some proteins are probably more directly involved in TM formation, the structure and stability of TMs are also affected more indirectly by pathways, which control the biogenesis of lipids and/or cofactors, and, e.g., mutants defective in synthesis of chlorophyll or of the membrane lipid phosphatidylglycerol (PG) have severely reduced TM systems [17,18,19,20].

Molecular chaperones of the Hsp70 family are involved in multiple cellular processes, such as folding of newly synthesized proteins, protein disaggregation, prevention of protein misfolding, protein transport, or the control of regulatory protein functions [21]. The thus far best characterized Hsp70 chaperone is the DnaK protein of the bacterium *Escherichia coli* [22]. In cyanobacteria, at least two DnaK proteins, DnaK2 and DnaK3, are highly conserved, and most cyanobacteria contain an additional DnaK1 protein as well as further DnaK-like proteins [15,23,24]. While cyanobacterial genomes typically encode several DnaK chaperones together with multiple DnaJ (Hsp40) proteins, which serve as DnaK co-chaperones, the physiological function of this DnaK-DnaJ network in cyanobacteria is essentially not understood. In recent years, the physiological roles of individual DnaK and DnaJ proteins have been analyzed to some extent in the cyanobacteria *Synechococcus* sp. PCC 7942 and *Synechocystis* [16,24,25,26]. In *Synechocystis*, three DnaK proteins are expressed together with at least seven DnaJ proteins [15,25]. The two *dnaK* genes *dnaK2* and *dnaK3* are essential in *Synechocystis*, but not *dnaK1* [15]. The DnaK2 protein has been classified as the canonical DnaK protein involved in cellular stress responses, and DnaK2 most likely functions together with Sll0897, the only type I DnaJ protein expressed in *Synechocystis* [24,25]. In line with this, deletion of the *sll0897* gene resulted in a heat-sensitive phenotype [25]. However, interactions with other DnaJ proteins cannot be excluded, and in fact, the DnaK2 protein interacts and cooperates with the type II J protein DnaJ2 in *Synechococcus* sp. PCC 7942 [27].

In contrast to the remaining *dnaJ* genes, the *dnaJ* gene *sll1933* (*dnaJ3*) could not be deleted in *Synechocystis*, indicating that the encoded DnaJ3 protein is essential [25]. The *dnaK3* and *dnaJ3* genes are organized in a conserved gene cluster in cyanobacteria, and a functional interaction of DnaK3 with DnaJ3 is assumed [28] DnaK3- and DnaJ3-homologs are encoded in essentially all cyanobacterial genomes, except in *Gloeobacter violaceus* PCC 4721, a cyanobacterium that lacks TMs [29,30]. Based on this observation it has been suggested that the physiological function of both proteins might be linked to TMs, and consequently, DnaK3 and DnaJ3 were suggested to be involved in the biogenesis and/or maintenance of TMs [16,25,31]. The DnaK3s of both *Synechococcus* and *Synechocystis* co-purify with membranes, and the unique DnaK3 C-terminus has been implicated to mediate tight membrane binding of DnaK3 in *Synechocystis* [15,31]. However, what might be the function of DnaK3 at TMs?

The function of a cyanobacterial DnaK3 has recently been linked to the PS II reaction center protein D1 [16], the main target of stress-induced damage in the photosynthetic electron transport chain, which is constantly degraded and replaced by newly synthesized proteins in a PS II repair cycle [32,33]. Furthermore, a Hsp70 chaperone is involved in the biogenesis, protection and/or repair of PS II complexes in chloroplasts [34,35]. Based on these observations we hypothesized that the physiological functions of DnaK proteins might have diverged in cyanobacteria, and DnaK3 potentially is specifically involved in biosynthesis/maintenance of TM complexes involved in photosynthesis.

In the present study, we have analyzed the role of the Hsp70 protein DnaK3 in TM maintenance in the cyanobacterium *Synechocystis* sp. PCC 6803. Expression of DnaK3 is light-regulated. Reduction of the cellular DnaK3 content resulted in decreased PS and phycobilisome (PBS) contents, a lowered PS I-to-PS II ratio, a generally reduced photosynthetic activity as well as disturbed PS II activity at elevated light conditions. The observation that the PS II activity is affected after photoinhibition in a mutant strain, where the cellular DnaK3 content is reduced, and the comparison of the mutant strain with *Synechocystis* wt suggests a specific function of DnaK3 in PS II protection and/or repair. However, based on the here presented data its activity must be wider. Thus, our findings support the assumption that DnaK3 is involved in biogenesis and/or maintenance of TM-localized electron transfer complexes in cyanobacteria.

## 2. Materials and Methods

### 2.1. Growth Conditions

A glucose-tolerant *Synechocystis* sp. PCC 6803 wild type (wt) and the merodiploid *dnaK3* (*sll1932*) knock-down (KD) strain [15] were cultivated photomixotrophically at 30 °C in liquid BG11 medium [36] supplemented with 5 mM glucose. Kanamycin (80 µg/mL) was added in case of the *dnaK3*KD strain. The cultures were aerated with air enriched with 2% CO_2_ and grown under fluorescent white light at a light intensity of 20 (LL, low light) or 120 (HL, high light) µmol/m^2^ s, respectively. To determine growth rates, the strains were initially adjusted to an OD_750_ of 0.05 in BG11 medium, containing 5 mM glucose, and growth was followed by monitoring OD_750_. For LAHG cultures, *Synechocystis* cells were grown in a dark cabinet for at least two weeks, during which the cultures were diluted at least five times in fresh medium, as described previously (Barthel et al., 2013).

### 2.2. SDS-PAGE and Immunoblot Analysis

*Synechocystis* cells were harvested in the exponential growth phase at an OD_750_ below 2.0. Cell pellets were resuspended in buffer (50 mM HEPES, pH 7.0, 25 mM CaCl_2_, 5 mM MgCl_2_, 10% (v/v) glycerol) and a proteinase inhibitor mix (Sigma Aldrich) was added at a 1:1000 dilution. Cells were broken with glass beads (0.25–0.5 mm diameter) in a beadbeater. Unbroken cells and glass beads were removed by centrifugation at 1600 g and the respective protein concentrations were determined by three independent Bradford assays. After addition of SDS sample buffer and heating at 65 °C for 15 min, cell extracts were loaded on an 8% polyacrylamide gel and proteins were separated by SDS gel electrophoresis. Subsequently, proteins were transferred to a polyvinylidene difluoride membrane, using a wet electroblotting system from Bio-Rad. The rabbit primary antibodies were used at 1:2000 (anti-L23 directed against the large ribosomal subunit protein L23 encoded by *sll1801*, Gramsch laboratories, Schwabhausen, Germany), 1:1000 (anti-DnaK1, anti-DnaK2 and anti-DnaK3 [15], anti-PsaA/PsaB [37]) or 1:100 (anti-PsbA [38]) dilutions, respectively, whereas the goat anti-rabbit secondary antibody (Sigma Aldrich) was diluted 1:10,000. PsbA/D1-HRP antibodies were obtained from Agrisera and used in 1:15,000 dilution. To visualize the protein bands, membranes were incubated with the enhanced chemiluminescence kit from Pierce. Each immunoblot analysis has been repeated at least three times.

### 2.3. Complete Deletion of DnaK3 in Synechocystis Cells Grown under LAHG Conditions

To test whether DnaK3 is dispensable in the dark, the *dnaK3*KD strain [15] was cultivated in liquid BG11 medium under LAHG conditions and diluted if necessary. During each dilution step, the concentration of kanamycin was enhanced in the growth medium from 80 to 275 µg mL^−1^. To check whether the strain was completely segregated, genomic DNA was isolated and analyzed by PCR using the primers NtdnaK3check (5’-gtttttagaagcggagaaagtgg-3´) and CtdnaK3check (5´-cctttgggttggaaaccattgg-3´).

### 2.4. Cell Number and Chlorophyll Concentration Determination

Cell numbers were counted with a light microscope using a Thoma counting chamber. Chlorophyll concentrations were determined photometrically after methanol extraction [39].

### 2.5. Electron Microscopy

To study the cell morphology of the different *Synechocystis* strains, cell pellets obtained from a 10 mL cell suspension were washed and resuspended in buffer (50 mM KH_2_PO_4_, pH 7). Ultrastructural investigations were performed as described previously [37]. The number of thylakoid layers per cell was determined, evaluating more than 200 individual cells of wt and the DnaK3 depleted *Synechocystis* strain, respectively.

### 2.6. Absorbance and Low Temperature (77K) Fluorescence Spectra

Absorbance spectra of whole cells were recorded using a Perkin-Elmer Lambda 25 spectrophotometer equipped with an integrating sphere. Cell suspensions were adjusted to a constant value of 300,000 cells mL^−1^. Ratios of cyanobacterial chromophores were determined using the absorption ratio at 625/680 (phycocyanin/chlorophyll) or at 490/440 (carotenoids/chlorophyll).

Low-temperature (77 K) fluorescence emission spectra were recorded using an Aminco Bowman Series 2 spectrofluorimeter. Cultures were adjusted to a chlorophyll concentration of 3 µg·mL^−1^ in BG11 medium and frozen in liquid nitrogen. Chlorophylls were excited at 435 nm and phycobilisomes (PBs) at 580 nm. Fluorescence emission was recorded from 630 to 760 nm.

### 2.7. Oxygen Evolution

Oxygen production of the cell suspensions was determined in the presence of 500 µM phenyl-p-benzoquinone (PPBQ) using a fiber-optic oxygen meter (PreSens) under actinic light (600 µmol photons m^−2^·s^−1^). Prior to the measurement, the cultures were adjusted to a chlorophyll concentration of 3 µg·mL^−1^ in BG11 medium. For experiments in presence of a protein synthesis inhibitor, 100 µg·mL^−1^ lincomycin was added prior to illumination (1500 µmol photons m^−2^·s^−1^).

### 2.8. Chlorophyll Fluorescence Induction Curves

Cultures were adjusted to a chlorophyll concentration of 3 µg·mL^−1^ in BG11 medium, and subsequently fluorescence induction curves were recorded at room temperature, using a Dual-PAM-100 measuring system equipped with Dual-E and DUAL-DR modules (Heinz Walz GmbH). During the initial dark phase, background fluorescence was probed by weak measuring light (0.024 µmol photons m^–2^·s^–1^) and after 40 s fluorescence was induced by switching on red actinic light (95 µmol photons m^–2^·s^–1^). Saturating pulses (600 ms, 10.000 µmol photons m^–2^·s^–1^) were applied once during the dark phase and at 30 s intervals during the light phase, to obtain minimal (F_0_) and maximal (F_m_ and F_m´_) fluorescence values [40,41]. The coefficient of photochemical quenching of the PS II Chl fluorescence (qP) was calculated using the software routine for light induction measurements (qP = (Fm-Fm’)/(Fm-Fo’)) after 250 s illumination with red actinic light.

### 2.9. P_700_ Re-Reduction Kinetics

Re-reduction kinetics were recorded using a Dual-PAM-100 measuring system. P_700_ was first reduced by 10 sec far-red and then oxidized by a 20 ms saturation light pulse (10.000 µmol photons m^–2^·s^–1^). 15 individual re-reduction curves were recorded, averaged, and fitted with single exponential functions to determine decay halftimes (t_1/2_). Prior to the measurement, the different cultures were adjusted to a chlorophyll concentration of 3 µg·mL^−1^ in BG11 medium.

## 3. Results

### 3.1. DnaK3 Synthesis is Light-Induced and Essential in the Dark

**Figure 1:**
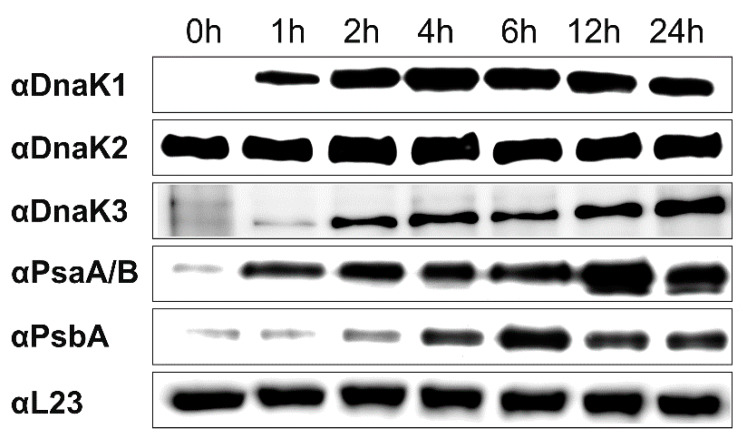
Light-dependent accumulation of DnaK1, 2, and 3. Dark-adapted *Synechocystis* cultures were shifted into the light (0–24 h). Cell extracts (20 µg protein) were analyzed at different time points via immunodetection, using anti-DnaK1, 2, or 3 antibodies as well as antibodies directed against PS I (PsaA/B) and PS II (PsbA) core subunits or the ribosomal protein L23 (loading control).

The *Synechocystis dnaK2* and *dnaK3* genes are essential in the light [15], and the DnaK1-3 proteins were detected by Western blot analyses in *Synechocystis* cells grown under constant illumination [15]. However, when *Synechocystis* cells were grown in the dark under LAHG conditions, the DnaK2 protein, but not DnaK1 and DnaK3, were detectable (Figure 1, 0 h). Yet, when dark-adapted cells were shifted into the light, the DnaK2 level did not substantially alter, whereas the DnaK1 level quickly increased until two hours after shifting the cells into the light. DnaK3 was detectable already after one hour, and its cellular content increased steadily. Thus, the synthesis of DnaK1 and DnaK3 clearly is triggered by light in *Synechocystis*.

Since DnaK1 is not essential for the viability of *Synechocystis* cells [15], we focused our subsequent analyses on DnaK3.

**Figure 2:**
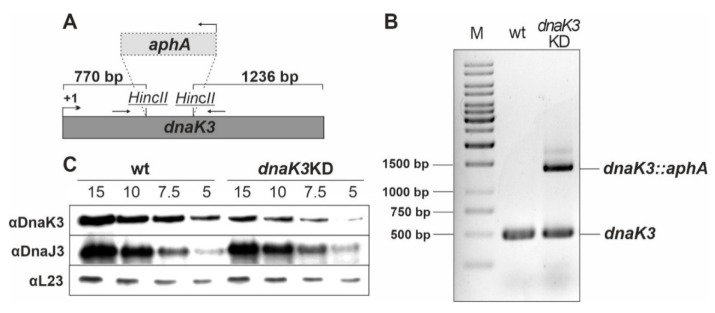
Deletion of *dnaK3* in the dark and the DnaK3 content in the *Synechocystis dnaK3*KD strain. (**A**) In the *Synechocystis dnaK3*KD strain [15], the *dnaK3* gene was disrupted by insertion of a kanamycin resistance cassette (*aphA* gene). (**B**) The *dnaK3* gene locus of wt and *dnaK3*KD cells grown in the dark was analyzed via PCR using genomic DNA as a template, and the PCR products were loaded on a 1.5% agarose gel together with a molecular size marker (M). Fragments of about 500 bp and 1500 bp represent the wt and the *dnaK3* gene interrupted by a kanamycin resistance cassette (*aphA*), respectively. (**C**) The relative DnaK3 content in the *dnaK3*KD strain was determined by immunoblot analysis. Cell extracts prepared from the wt and *dnaK3*KD strains, respectively, were loaded on a SDS-polyacrylamide gel in descending protein concentrations (15 µg to 5 µg) followed by a Western blot analysis using α-DnaK3, α-DnaJ3 and α-L23 (loading control) antibodies.

As DnaK3 is essential in the light [15], the observation of a light-induced DnaK3 synthesis indicated that DnaK3 might be dispensable in the dark. Therefore, we next attempted to completely delete the *Synechocystis dnaK3* gene in cells grown in the dark under LAHG conditions. Yet, even after more than half a year of cultivation under LAHG conditions and increasing the kanamycin concentration in the growth medium up to 275 µg·mL^−1^, a fragment corresponding in size to the wild type (wt) *dnaK3* gene was always detected via PCR in the *dnaK3* knock-down (*KD)* strain in addition to the *dnaK3* gene disrupted by the kanamycin resistance (*aphA*) cassette (Figure 2A,B). As *Synechocystis* contains multiple identical genome copies, this result indicates that some, but not all, of the genomic *dnaK3* copies were deleted in the mutant strain. Thus, DnaK3 likely is essential not only in the light but also in the dark under LAHG conditions.

Yet, we recently showed that expression of *dnaJ3* [25], which is organized in a gene cluster together with *dnaK3*, is essential in *Synechocystis*, and thus deletion of *dnaK3* might have affected the expression of *dnaJ3*. To assess this potential polar effect, we also quantified the amount of the DnaJ3 protein in the *dnaK3*KD strain (Figure 2A). Since the DnaJ3 level was not decreased compared to the wt, we concluded that insertion of the *aphA* cassette into the *dnaK3* gene locus did not dramatically affect the expression of *dnaJ3*. Nevertheless, a polar effect on expression of *dnaJ3* cannot be completely excluded.

To quantify the relative cellular DnaK3 content in the *dnaK3*KD strain, total cellular extracts of the wt and the KD strain were analyzed via Western blots (Figure 2C). The intensity of each band was quantified using the Image J software and divided by the quantity of cellular extract loaded. Based on this analysis, the DnaK3 content was decreased by about 60% ± 10% in the *dnaK3*KD strain compared to the wt.

### 3.2. Reducing the DnaK3 Content Affects Cell Growth under Heat Stress Conditions

**Figure 3:**
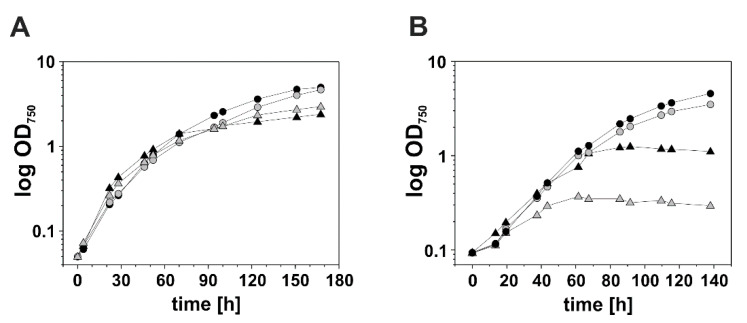
Growth of *Synechocystis* wt and the *dnaK3*KD mutant strain at different growth conditions. *Synechocystis* wt (black) and *dnaK3*KD mutant (gray) cells were grown at (**A**) moderate temperature (30 °C) or (**B**) elevated temperature (42 °C) under low light (circle) or high light (triangle) conditions. Cells were adjusted to OD_750_ = 0.05 in BG11 medium containing 5 mM glucose and cell growth was followed over time by measuring the OD_750_.

Next, we tested whether reducing the DnaK3 content affects growth of the mutant strain under low (LL) or high light (HL) growth conditions, respectively (Figure 3A). The *dnaK3*KD and the wt cells had comparable doubling times of 11.2 h ± 0.1 (wt) and 11.1 h ± 0.2 (*dnaK3*KD), and of 8.1 h ± 0.5 (wt) and 9.4 ± 1.2 (*dnaK3*KD) under LL and HL growth conditions, respectively. Thus, reducing the cellular DnaK3 content does not severely affect the growth of *Synechocystis* cells, at least not under standard laboratory growth conditions. Subsequently, growth of the *dnaK3*KD strain was tested under various stress conditions (involving low pH, low temperature, oxidative and osmotic stress; data not shown), but solely increasing the temperature to 42 °C resulted in an obvious growth defect of the mutant strain, with doubling times of 26.4 ± 3.5 h (*dnaK3*KD) and 17.9 ± 0.4 h (wt) (Figure 3B). This observation classifies DnaK3 as a traditional Hsp70 involved in heat-stress responses.

### 3.3. The dnaK3KD Strain Has a Reduced Pigment Content

Photosynthesis is one of the most temperature-sensitive processes in phototrophic organisms and the photosynthetic activity is further impaired when heat-stress is combined with HL [42,43]. Thus, it was well possible that reducing the DnaK3 content affects photosynthetic processes in *Synechocystis*.

As expression of *dnaK3* is light-controlled (Figure 1), we subsequently analyzed the pigment content of the wt and *dnaK3*KD strains after cultivation under LL and HL conditions, respectively. Adaptation of *Synechocystis* cells to HL conditions is typically accompanied by a reduction in the cellular amount of the two PSs, a decreased PS I-to-PS II ratio and a reduced chlorophyll (Chl) content per cell [44,45].

**Figure 4:**
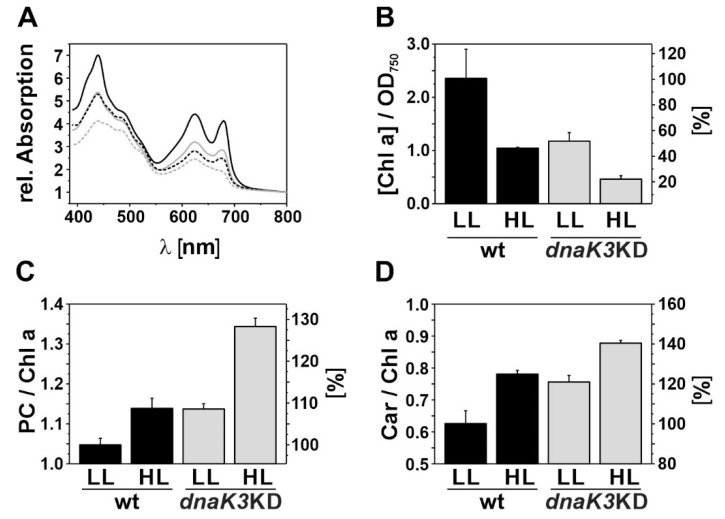
Pigment content and pigment ratios. (**A**) Absorbance spectra of *Synechocystis* wt (black) and *dnaK3*KD (gray) cells (300,000 cells) grown under LL (solid line) or HL (dashed line) conditions. (**B**) The chlorophyll content per OD_750_ was determined as described in “Material and Methods”. (**C**) The ratio of PC to Chl was determined as the ratio of the absorptions at 625 and 680 nm. (**D**) The ratio of Car to Chl was determined as the ratio of the absorption at 490 and 440 nm. Error bars represent standard deviation from three independent experiments.

An overall reduction of the pigment content was observed under HL growth conditions when equal amounts of cells were analyzed (Figure 4A). The Chl content was reduced to about half (Figure 4B) and the relative content of plastocyanine (PC) (Figure 4C) and carotenoids (Car) (Figure 4D) were both increased. It has to be noted that while the contents of Chl, PC and Car were decreased under HL growth conditions (Figure 4A), the PC/Chl as well as the Car/Chl ratios were increased in the wt strain, due to the more severely decreased Chl content (Figure 4A,B). Even though light scattering could have contributed to some extent to the determined (absolute) absorbance values used in these analyses, these data clearly show the ability of the wt to reduce the overall pigment content and to adapt it to HL conditions.

Similarly, the *dnaK3*KD strain adapted to changing light conditions and reduced its pigment content as expected when grown under HL conditions. However, the *dnaK3*KD strain exhibited a severely reduced pigment content already when grown under LL conditions, and the Chl content as well as the pigment ratios were very similar to the ones observed when the wt was grown under HL conditions (Figure 4).

**Figure 5:**
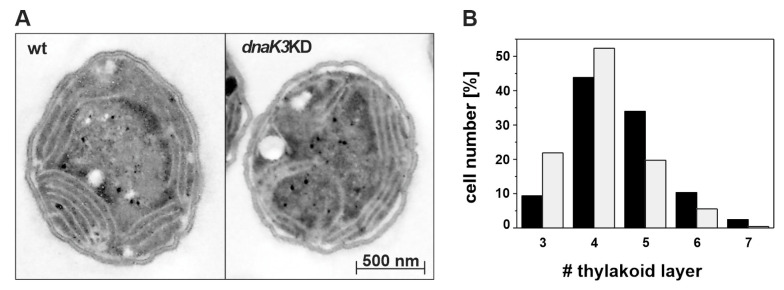
Reducing the cellular DnaK3 content results in fewer thylakoid layers. (**A**) Representative electron micrographs of *Synechocystis* wt and *dnaK3*KD mutant cells cultivated under LL (low light) conditions. (**B**) Cells of the *dnaK3*KD mutant strain (gray) had four thylakoid layers on average, whereas wt cells (black) showed four to five layers and a higher appearance of six and seven layers of TM pairs. Per strain, at least 200 individual cells were counted.

Besides the obvious differences in pigmentation, the TM structure was mostly unaffected, and we only observed a slightly reduced number of TM pairs in the mutant strain when the ultrastructure of *Synechocystis* grown under LL growth conditions was analyzed via electron microscopy (Figure 5).

### 3.4. Reducing the DnaK3 Content Results in an Altered PS I-to-PS II Ratio

**Figure 6:**
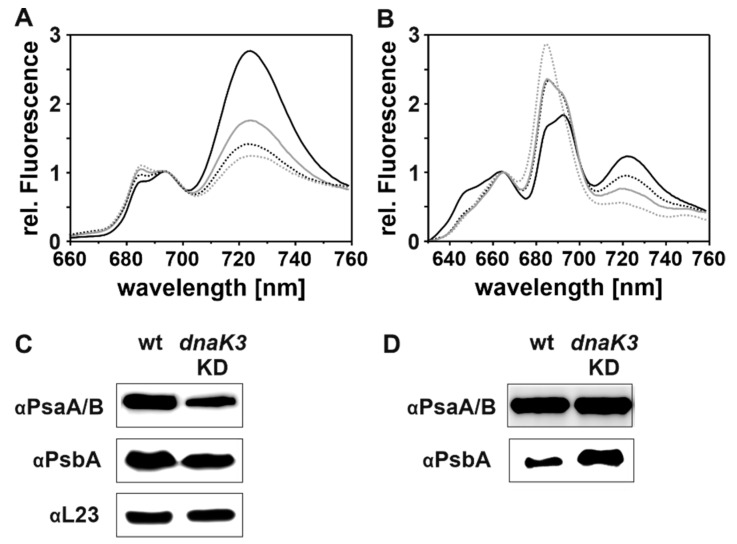
Reduction of the cellular DnaK3 content results in a decreased PS content and a lowered PS I-to-PS II ratio. (**A**) 77 K fluorescence emission spectra of wt (black) and *dnaK3*KD (gray) cultures grown under LL (solid line) and HL (high light) (dashed line) conditions. The spectra were normalized at 695 nm. λ_Ex_ = 435 nm (**B**) 77 K fluorescence emission spectra of wt (black) and *dnaK3*KD (gray) cultures cultivated under LL (solid line) and HL (dashed line) conditions. The spectra were normalized at 665 nm. λ_Ex_ = 580 nm. (**C**,**D**) Immunoblot analysis of the content of PS I and PS II core subunits (PS I: PsaA/B; PS II: PsbA) in wt and *dnaK3*KD cells grown under LL conditions. Samples were normalized to (C) protein (25 µg) or (D) chlorophyll (0.6 µg). L23 is the loading control.

Next, the relative amounts of PS II and PS I in the DnaK3 reduced strain were determined via 77 K fluorescence spectroscopy (Figure 6A). Upon chlorophyll excitation at 435 nm, characteristic fluorescence emission maxima were detected at 721 nm (PS I), at 684 nm (CP43, PS II) and 693 nm (CP47, PS II).

*Synechocystis* wt cells grown under HL conditions showed a decreased PS I-to-PS II ratio compared to LL-adapted cells (Figure 6A), which is a well-documented long-term adaptation to HL [44,46,47]. In contrast, *dnaK3*KD cells had a considerably decreased PS I-to-PS II ratio already under LL growth conditions. This finding is also supported by a Western blot analysis. When an identical quantity of protein was loaded, the Western blot shows that PS core subunits PsaA/B (PS I) and PsbA (PS II) are less abundant in the *dnak3*KD strain (Figure 6C). When the cell extracts were normalized based on the Chl concentration (Figure 6D), no difference in the band intensity was observed in case of PsaA/B, since in *Synechocystis* about 85% of the Chl is bound to PS I (assuming a PS I/PS II ratio of 2.5 [48], 96 chlorophylls per PS I [49], and 35 chl per PS II [50]). However, the PsbA band was more pronounced in the mutant strain when compared to the wt, which further supports the decreased PS I-to-PS II ratio in this strain. The decreased PS I-to-PS II ratio decreases even further when cells were shifted into HL (Figure 6A).

However, at LL as well as at HL conditions, an increased relative fluorescence emission was observed at 684 nm (λ_ex_ = 435nm) in the mutant strain (Figure 6A). This fluorescence emission maximum originates from PS II as well as from the PBSs terminal emitter LCM [51], and thus indicates an increased relative phycobiliprotein content, as already observed in the absorbance measurements (Figure 4A,C). Yet, the increased PBSs fluorescence emission at 684 nm is solely observed when PBSs are uncoupled and do not transfer the harvested light energy to the PSs [51,52]. Thus, to next assess energy transfer from PBSs to PS II, phycobiliproteins were excited at 580 nm and energy transfer to PS II was followed. When PBSs are coupled to PS II, light energy harvested by the PBS is transferred to PS II, resulting in quenching of the PBS fluorescence [53,54]. As can be seen in Figure 6B, upon PBS excitation an increased fluorescence emission at 684 nm (PBSs plus PS II) was observed but not at 693 nm (PS II) (Figure 6B). Thus, the *dnaK3*KD strain indeed contains an increased amount of uncoupled PBSs compared to the wt.

The decreased fluorescence emission at 721 nm results from the decreased PS I content (Figure 6A) and most likely not from light-dependent energy distribution via state transitions, which is supposed to be physiologically important solely under LL growth conditions [55,56].

Taken together, the fluorescence spectra and the Western blot analyses reveal that the mutant has a generally decreased PS content, with a decreased PS I-to-PS II ratio and an increased amount of uncoupled phycobiliproteins. However, the mutant strain still adjusts the PS I-to-PS II ratio to changing light conditions, as observed for the wt strain.

### 3.5. The Photosynthetic Activity is Impaired in the DnaK3 Depleted Strain

**Figure 7:**
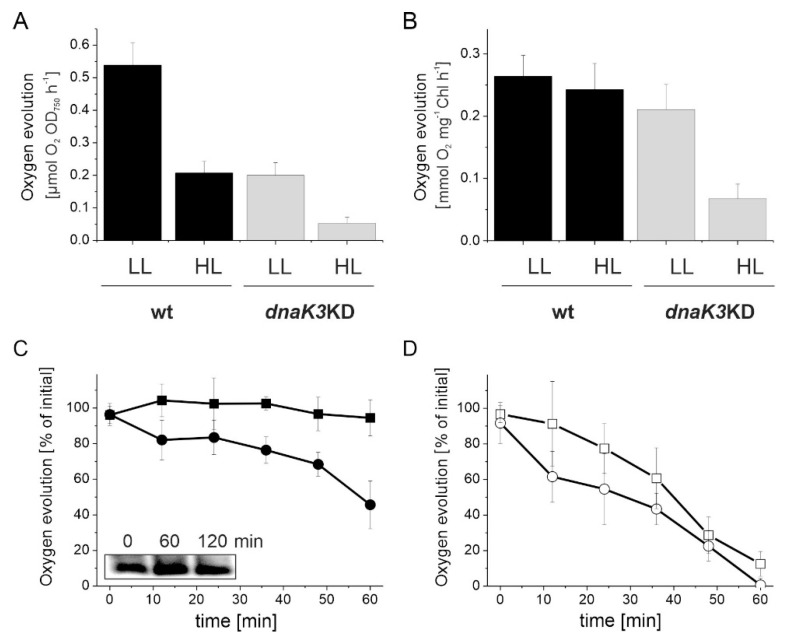
Oxygen evolution rates and relative PS II content of wt and *dnaK3*KD *Synechocystis* cells. (**A**,**B**) wt and *dnaK3*KD cells were grown under LL and HL conditions, respectively, and oxygen evolution rates were determined per OD_750_ (**A**) or Chl (**B**). (**C**,**D**) wt (square) and *dnaK3*KD (circle) cells were exposed to extreme high light (eHL) conditions (1500 µmol photons m^−2^·s^−1^) either in absence (**C**) or presence (**D**) of lincomycin (100 µg·mL^−1^) and thereafter cultured under LL conditions for recovery. Oxygen evolution was measured using 500 µM phenyl-p-benzoquinone (PPBQ) as an electron acceptor at PS II. The recovery rate is given by the slope of a linear regression under LL conditions. Noteworthy, no other antibiotics were present in these experiments. Inlet in (C): Immunoblot analysis of the D1 content in the *dnaK3*KD strain after photoinhibition (time 0). Cell extracts with identical Chl contents (0.4 µg) were analyzed. (Error bars represent standard deviation from three independent experiments).

Next, the photosynthetic activity of the mutant strain with a reduced DnaK3 content was studied in greater detail. By measuring oxygen evolution rates in presence of PPBQ, the activity of PS II can be specifically determined (Figure 7A,B). When adapted to HL growth conditions, the O_2_ evolution rate per cell (OD_750_) was reduced in the wt strain compared to LL growth conditions (Figure 7A), in line with the observation that the light-harvesting capacity is generally reduced in cyanobacterial cells under HL conditions [57]. In contrast to wt cells, the *dnaK3*KD strain showed a dramatically decreased O_2_ evolution rate already under LL conditions, when compared to the wt, and the activity decreased even further under HL conditions (Figure 7A). However, when the O_2_ evolution rates were normalized to the Chl content, the O_2_ evolution rate remained essentially stable in the wt, regardless of the light conditions (Figure 7B). In contrast, the O_2_ evolution rate was only marginally lower for the mutant strain under LL growth conditions than for the wt, but dramatically decreased under HL growth conditions. Thus, in contrast to the wt, the decreased O_2_ evolution in the mutant strain is not only a consequence of the decreased cellular Chl content (Figure 4B), since the O_2_ evolution rate was also drastically decreased when the measurements were normalized to the Chl content (Figure 7B).

To test whether reducing the DnaK3 content somehow impairs PS II repair, we next determined O_2_ evolution rates under extreme HL conditions (1500 µmol photons m^−2^·s^−1^) in presence or absence of lincomycin, a protein synthesis inhibitor that has already been successfully used to block the PS II repair cycle in *Synechocystis* [58,59]. In absence of lincomycin, the wt strain did not show any changes in the PS II activity under constant extreme HL illumination, i.e., the wt cells harbor an effective PS II repair cycle (Figure 7C). However, in presence of lincomycin, the PS II activity constantly decreased when cells were illuminated with extreme HL (Figure 7D). The decreasing PS II activity, i.e., an impaired PS II protection and/or repair, can be observed for both the wt and the *dnaK3*KD strain in presence of lincomycin (Figure 7D). However, in absence of lincomycin, the PS II activity was already lower in the *dnaK3*KD than in the wt strain after 10 min of illumination and constantly decreased further to about 50% after 1 h of illumination, whereas the wt activity remained about constant (Figure 7C). Thus, PS II repair clearly is severely impaired in *dnaK3*KD cells, albeit the amount of expressed D1 protein did not alter (inlet in Figure 7C). Thus, PS II protection and/or repair is affected especially under light-stress conditions.

**Figure 8:**
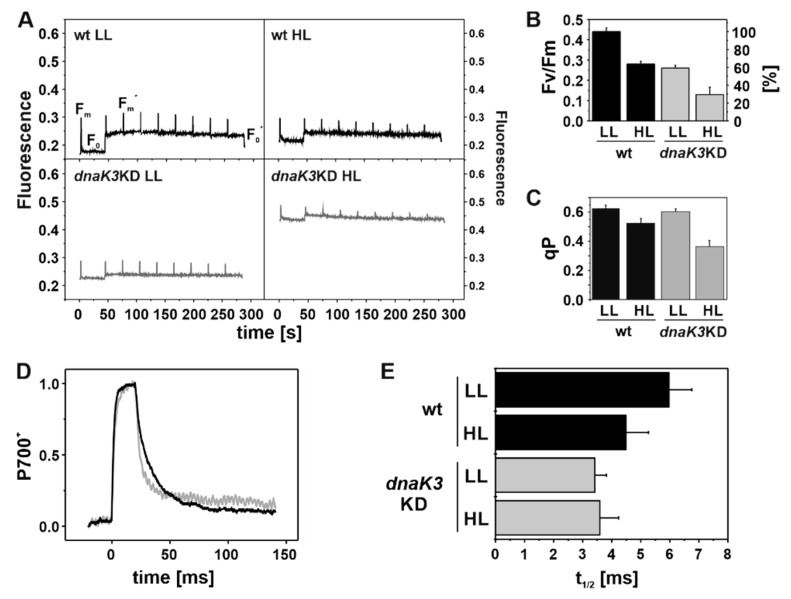
PS II activity and P_700_ re-reduction kinetics. (**A**) Light/dark induction curves were recorded by measuring the pulse amplitude modulated (PAM) fluorescence of wt (black) and *dnaK3*KD cells (gray) grown under LL or HL conditions, respectively. After 40 s of measuring light, the actinic red light was switched on to determine minimal fluorescence values (F_0_, F_0_^´^). Pulses of saturating light were applied once during the dark phase and in 30 s intervals during the light phase, to obtain maximal (F_m_ and F_m_^´^) fluorescence values. (**B**) Maximal PS II photosynthetic activity of the wt (black) and the *dnaK3*KD strain (gray). (**C**) The coefficient of photochemical quenching of PS II Chl fluorescence (qP) in wt (black) and *dnaK3*KD cells (gray). (B, C) Error bars represent standard deviation from at least four independent experiments. (**D**) P_700_^+^ re-reduction kinetics of wt (black) and *dnaK3*KD (gray) cells grown under LL conditions. A saturation pulse of 10,000 µmol photons was given for 20 ms to completely oxidize P_700_. The following fluorescence decrease illustrates re-reduction of P_700_^+^ in the dark. At least ten traces were averaged and normalized. 1 represents completely oxidized and 0 completely reduced P_700_. (**E**) Re-reduction halftimes were determined via fitting the decay curves of the wt (black) and the *dnaK3*KD mutant strain (gray) with single exponential functions. Error bars represent standard deviation from at least three independent experiments.

The photochemical efficiency of PS II can be specifically assessed using pulse amplitude modulated (PAM) fluorescence measurements. Therefore, dark/light induction curves were recorded (Figure 8A). A minimal fluorescence (F_0_) is visible due to the measuring light, which, however, is not strong enough to stimulate photosynthetic electron transfer. Subsequently, a pulse of intense white light is given to reduce all PS II reaction centers, resulting in maximal fluorescence (F_m_). The parameter F_v_/F_m_ (F_v_ = F_m_ − F_o_) is used to describe the maximal photochemical efficiency (Figure 8B) [60].

After switching on actinic light, an increased background fluorescence was detected, and PS II centers became photosynthetically active (Figure 8A). Saturating light pulses resulted in a lowered F_m_´ compared to the maximal fluorescence F_m_ measured in the dark, due to non-photochemical quenching processes [61]. An apparent increase of the absolute F_0_ background fluorescence was measured for wt cells grown under HL conditions compared to LL and for the *dnaK3*KD cells (grown under either condition), indicating a more reduced plastoquinone (PQ) pool (Figure 8A). However, determining F_0_ and F_0_´ is somewhat problematic in cyanobacteria, as the PBS fluorescence can in part also contribute to the determined F_0_ fluorescence value [62], and thus discussion of solely F_0_ values is difficult. Hence, we also present the normalized coefficient of photochemical quenching of PS II Chl fluorescence (qP), which is not significantly biased by PBSs fluorescence [63]. qP is defined as 1 in the dark-adapted state and may decrease to 0 when all PS II centers are closed. In line with Figure 7B, in the wt strain slightly less PS II centers are open under HL conditions compared to LL. In the mutant strain, qP is similar to the wt under LL conditions, yet the value was dramatically decreased when *dnaK3*KD cells were grown under HL conditions, indicating an increased amount of closed PS II centers. Thus, the *dnaK3*KD strain can hardly cope with high light treatment. This observation is in excellent agreement with the determined O_2_ evolution rates (Figure 7), showing an impaired PS II protection and/or repair cycle.

P_700_^+^ re-reduction measurements allow determining the time needed to re-reduce the oxidized PS I reaction center P_700_^+^, which is not only affected by the PS I activity but also by the redox state of the electron transport chain (Figure 8D,E). The P_700_^+^ absorbance signal increased when a saturating light pulse, which completely oxidized P_700_, was given and subsequently decreased due to P_700_^+^ reduction by PC (Figure 8D). The halftime of the re-reduction kinetic was determined by fitting the changes in the absorbance signal with a single exponential function (Figure 8E). A faster re-reduction rate was observed for wt cells when cells were grown under HL compared to LL conditions, which likely originates from the reduced PS I content and the decreased PS I-to-PS II ratio (Figure 6 and Figure 8). In contrast, the *dnaK3*KD mutant strain had reduced re-reduction halftimes under both tested light conditions, and the halftimes were identical, regardless of the light condition (Figure 8E). The reduced re-reduction halftimes can be explained by a more reduced PQ-pool and thus nicely support the conclusions drawn from the results shown in Figure 6 and Figure 8. Together, these results demonstrate that the activity of PS II, but not of PS I, is impaired in the *dnaK3*KD mutant strain.

## 4. Discussion

Three different DnaK proteins are expressed in the cyanobacterium *Synechocystis* sp. PCC 6803. While two of the cyanobacterial DnaK proteins, DnaK2 and DnaK3, are essential, solely DnaK2 can be classified as a canonical Hsp70 protein, expression of which can largely alter under various stress conditions [24,64,65]. In contrast, the DnaK3 chaperone of *Synechocystis* has been suggested to be specifically involved in biogenesis and/or maintenance of TMs [16,25]. However, thus far this assumption was essentially exclusively based on the observations that (i) DnaK3 is attached to TMs and (ii) DnaK3 is encoded in all cyanobacterial genomes, except in *Gloeobacter violaceus*, the only cyanobacterium that does not contain an internal TM system [15,29].

Albeit the cellular DnaK3 content clearly is light-regulated (Figure 1), a basal DnaK3 level appears to be required for survival of *Synechocystis* cells not only in the light but also under LAHG conditions (Figure 2), where cells still have rudimentary TMs. Based on the CyanoExpress database the *dnaK3* transcript level does not appear to adjust to changing light conditions in *Synechocystis* [66] or in *Synechococcus* sp. PCC 7942 [16]. Thus, (light-dependent) DnaK3 synthesis likely is post-transcriptionally regulated, as common in cyanobacteria [67,68].

A general decrease in the PS and PBS content per cell as well as a selective down-regulation of PS I is crucially involved in the adaptation of *Synechocystis* cells to HL conditions [46,67]. All these (expected) adjustments were observed when the *Synechocystis* wt strain was shifted from LL to HL growth conditions (Figure 6 and Figure 7). Also, in case of the mutant strain, typical HL-adaptation processes were observed, although the light-induced changes were far less pronounced, since the mutant strain already exhibited characteristics of an HL-stressed strain under LL growth conditions. While in the *dnaK3*KD strain the relative PS I content was reduced and the PS I-to-PS II ratio per cell was lower than in the wt (Figure 6), PS I appears to function normally when DnaK3 was depleted, because re-reduction of PS I was even faster in the mutant strain (Figure 8E), most likely due to the more reduced PQ-pool, which also results in a significant amount of the PBSs being detached from PS II (Figure 6B).

Yet, the activity of PS II was clearly reduced in the mutant strain (Figure 7 and Figure 8). A significant amount of PS II was inactive, potentially due to increased photodamage and/or impaired repair (Figure 7 and Figure 8). Thus, DnaK3 likely is involved in PS II biogenesis and/or repair. In line with this assumption, expression of the *Synechocystis dnaK3* gene was found being enhanced under UV-B stress [68]. Furthermore, the PS II core subunit D1 was proposed to be a substrate for DnaK3, which potentially guides the nascent polypeptide at the ribosome to the TM, where translation is completed [16]. The D1 protein is known to be especially susceptible to photodamage, and photodamaged D1 is rapidly degraded and replaced by newly synthesized protein to maintain a certain level of active PS II centers in cyanobacteria [33]. Thus, the here presented results clearly indicate that biogenesis and/or repair of PS II is impaired when the cellular DnaK3 content is reduced.

However, the observation that DnaK3 appears to be vital also in the dark (Figure 2), where PS II is inactive [7] and the finding that D1 is not essential for survival of *Synechocystis* under photoheterotrophic conditions [69] indicates that DnaK3 likely has additional physiological functions beyond PS II protection and/or repair. In cyanobacteria, TMs also contain the complexes of the respiratory e^−^-transfer chain [70], and the indications of an over-reduced PQ pool (Figure 6 and Figure 8) suggests that other proteins and protein complexes are also affected when the DnaK3 content is reduced. A broader implication of DnaK3 in TM biogenesis and maintenance, involving biogenesis and/or repair of multiple TM complexes would be a convincing explanation.
